# Loss of *PBRM1* rescues *VHL* dependent replication stress to promote renal carcinogenesis

**DOI:** 10.1038/s41467-017-02245-1

**Published:** 2017-12-11

**Authors:** Judit Espana-Agusti, Anne Warren, Su Kit Chew, David J. Adams, Athena Matakidou

**Affiliations:** 1 Department of Oncology, University of Cambridge, CRUK Cambridge institute, Cambridge, CB2 0RE UK; 2 Department of Pathology, Addenbrooke’s Hospital, Cambridge, CB2 0QQ UK; 3 Cancer Genome Project, Wellcome Trust Sanger Institute, Hinxton, CB10 1SA UK; 4 Translational Cancer Therapeutics Laboratory UCL Cancer Institute, London, WC1E 6DD UK; 5 Experimental Cancer Genetics, Wellcome Trust Sanger Institute, Hinxton, CB10 1SA UK; 6 Present Address: MedImmune, AstraZeneca, Cambridge, CB21 6GH UK; 7 Present Address: Centre for Genomics Research, IMED Biotech Unit, AstraZeneca, Melbourn, SG8 6HB UK

## Abstract

Inactivation of the *VHL* (Von Hippel Lindau) tumour suppressor has long been recognised as necessary for the pathogenesis of clear cell renal cancer (ccRCC); however, the molecular mechanisms underlying transformation and the requirement for additional genetic hits remain unclear. Here, we show that loss of VHL alone results in DNA replication stress and damage accumulation, effects that constrain cellular growth and transformation. By contrast, concomitant loss of the chromatin remodelling factor PBRM1 (mutated in 40% of ccRCC) rescues VHL-induced replication stress, maintaining cellular fitness and allowing proliferation. In line with these data we demonstrate that combined deletion of *Vhl* and *Pbrm1* in the mouse kidney is sufficient for the development of fully-penetrant, multifocal carcinomas, closely mimicking human ccRCC. Our results illustrate how *VHL* and *PBRM1* co-operate to drive renal transformation and uncover replication stress as an underlying vulnerability of all *VHL* mutated renal cancers that could be therapeutically exploited.

## Abstract (web summary)

Mutations in VHL have been linked to clear cell renal cancer, but the molecular mechanisms involved remain unclear. Here the authors generate a mouse model closely mimicking the human disease and show that VHL loss induces DNA replication stress that is rescued by the concomitant loss of PBRM1 permitting transformation.

## Introduction

Renal cancer is the twelfth commonest cancer worldwide, accounting for 338,000 new cases and 143,000 cancer-related deaths a year^1^. Around 50% of all patients develop metastatic disease and in this setting, renal cancer remains incurable with a median survival of around 28 months^2^. The development of effective treatment regimens has been hampered by a limited understanding of the basic molecular pathways underpinning renal cell carcinoma (RCC) carcinogenesis and the lack of genetically relevant animal models.

Clear cell RCC (ccRCC), the commonest histological subtype, is characterised by the inactivation of *VHL* tumour suppressor gene^3^. *VHL* is responsible for the ccRCC-predisposing syndrome, von Hippel-Lindau^4^ and is inactivated by either mutation or methylation in over 80% of sporadic cases^5–7^. The second allele is lost as a part of large 3p deletions, observed in ~90% of all cases^8–10^. Chromosome 3p loss and *VHL* mutation or methylation, are early, truncal events during RCC development^11^ and therefore considered crucial for renal transformation.

VHL is implicated in a number of cellular functions, disruption of any or all of which could contribute to carcinogenesis. These include mediation of adaptive responses to hypoxia (through activation of hypoxia inducible factors HIF1a and HIF2a), extracellular matrix assembly, ciliogenesis, microtubule stabilisation, senescence, and DNA repair^3, 12^. Current data, however, suggest that *VHL* loss alone, though necessary, is not sufficient for tumour initiation in the kidney. First, kidneys of patients with familial VHL disease typically contain many thousands of sites of biallelic *VHL* loss, with ccRCC arising relatively infrequently^13^. Second, large-scale sequencing efforts have shown that biallelic intragenic *VHL* mutations are not observed in sporadic ccRCC but instead one copy of the gene is always lost as a part of chromosome 3p deletion, simultaneously affecting a number of other genes^11, 14^. Third, deletion of *Vhl* or activation of its main downstream effectors (Hif1a/Hif2a) within the mouse kidney does not lead to tumour development^15–19^. These observations suggest the requirement of additional genetic events to drive transformation.

Recently, next-generation sequencing studies have identified the SWI/SNF chromatin remodeller *PBRM1* as the second most frequently mutated ccRCC gene^10, 14^. Concomitant loss of *VHL* and *PBRM1* is observed in up to 40% of all cases^14^ and phylogenetic analyses have suggested that *PBRM1* may act as a renal cancer driver as mutations are frequently acquired early^11^. Here we set out to ascertain the molecular interactions between VHL and PBRM1, and their potential to drive renal carcinogenesis in vivo. Our data show that loss of VHL alone induces replication stress and DNA damage accumulation, responses that curtail cellular proliferation and transformation. Significantly, the concomitant loss of PBRM1 rescues VHL-dependent replication stress, conferring a survival and proliferative advantage and thereby permitting renal carcinogenesis. Our study provides essential insights into the molecular mechanisms underpinning renal transformation and highlights replication stress as an underlying vulnerability of ccRCC.

## Results

### PBRM1 loss rescues VHL-induced replication stress

To identify interactions arising from the concurrent loss of VHL and PBRM1 within the context of normal cellular physiology, we generated primary mouse embryonic fibroblasts (MEFs) from either wild-type (WT), *Vhl* ^*fl/fl*^, *Pbrm1* ^*fl/fl*^ or double-mutant (*Vhl* ^*fl/fl*^;*Pbrm1* ^*fl/fl*^) conditional mice. Recombined MEFs are hereafter referred to as *Vhl* ^*−/−*^, *Pbrm1* ^*−/−*^ and *Vhl* ^*−/−*^;*Pbrm1* ^*−/−*^ (Supplementary Fig. 1a,b). To replicate physiological tissue conditions^20^ and avoid the well-documented sensitivity of MEFs to 21% oxygen^21, 22^, all experiments were performed at 5% O_2_. As both VHL and PBRM1 have been previously implicated in DNA repair and the maintenance of genome stability^12, 23–25^, pathways essential for tumour suppression, we decided to investigate their combined effect on these processes.

**Fig. 1:**
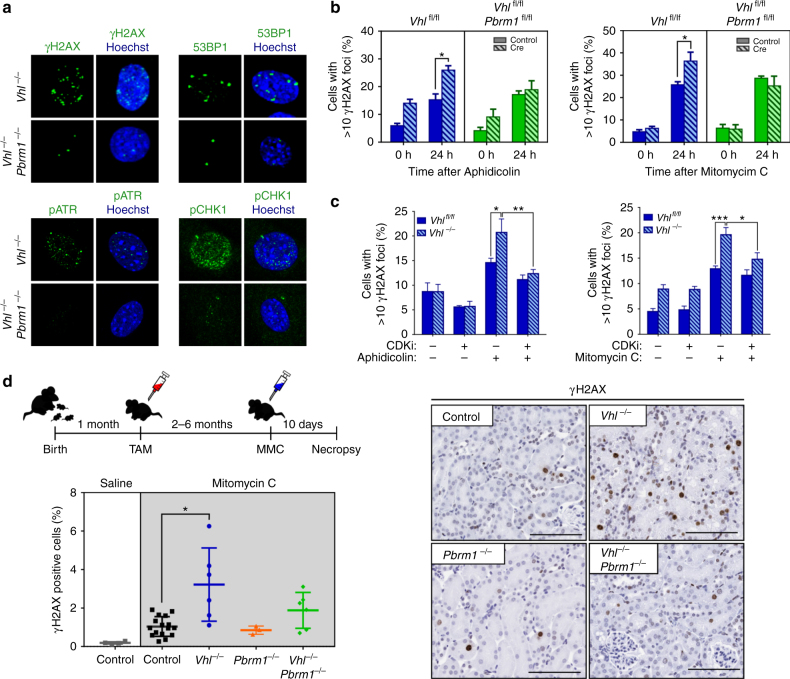
Loss of VHL induces replication stress rescued by concomitant loss of PBRM1. **a** Representative photos of immunofluorescence staining in day 20 recombined MEFs. **b**, **c** Quantification of γH2AX foci formation following treatment of MEFs as indicated (**b**, *n* (independent experiments) = 3; **c**, left, *n* (independent experiments) = 6; right, *n* (independent experiments) = 4). **d** Schematic of experimental design of in vivo Mitomycin C (MMC) experiment; renal γH2AX quantification following MMC and representative immunohistochemical photos (*n* (mice); Saline: control = 6; MMC: control = 16; *Vhl* ^*−/−*^ = 6; *Pbrm1* ^*−/−*^ = 3; *Vhl* ^*−/−*^ *;Pbrm1* ^*−/−*^ = 7). TAM: tamoxifen. Scale bars, 100 μm. **b**, **c** Graphs depict means ± s.e.m. (error bars); two-way ANOVA, Sidak’s correction. **d**, Graph depicts means ± s.d. (error bars); Kruskall–Wallis, Dunn’s correction. ***p < 0.001, **p < 0.01, *p < 0.05

Analysis of DNA damage markers demonstrated significant accumulation of γH2AX and 53BP1 foci in late (day 20 of culture) vs. early (day 8) *Vhl* ^*−/−*^ cells (Fig. 1a and Supplementary Fig. 1c), indicating accrual of DNA damage upon replication. Surprisingly, this phenotype was restricted to *Vhl* ^*−/−*^ cells with *Vhl* ^*−/−*^;*Pbrm1* ^*−/−*^ MEFs displaying no significant damage accumulation. This disparity was not attributable to differences in the proliferation or senescence rates between the two groups (Supplementary Fig. 1d,e), suggesting that the rescue of this *Vhl*-induced phenotype was mediated by loss of *Pbrm1*.

The accumulation of DNA damage markers in *Vhl*-deleted cells is indicative of the presence of replication stress. Replication stress broadly defines impediments in DNA replication, including stalling and collapse of replication forks and activation of the DNA damage response primarily mediated by the kinase ATR (ATM- and Rad3-related)^26^. Indeed, late passage *Vhl* ^*−/−*^ MEFs displayed significant activation of both ATR and its effector kinase CHK1 (Fig. 1a and Supplementary Fig. 1c). To investigate whether VHL loss induces replication stress, MEFs were assayed for hypersensitivity to mitomycin C (MMC) and aphidicolin (APH), agents which result in replication fork stalling. *Vhl* ^*−/−*^ cells displayed significantly higher levels of DNA damage markers upon treatment with these agents compared to non-recombined controls (Fig. 1b and Supplementary Fig. 2). This phenotype was rescued by concomitant treatment with cyclin-dependent kinase inhibitors to halt S-phase progression (Fig. 1c), suggesting the presence of a replication-dependent defect.

To ascertain the relevance of these observations within differentiated renal tubular epithelia (the presumed cell of origin of human ccRCC)^27–29^, we sought to generate genetically engineered mice with renal tubular specific deletion of *Vhl*, *Pbrm1* or both *Vhl* and *Pbrm1*. We have previously described the development of *Pax8CreER* ^*T2*^ transgenic mice^16^ that allow highly specific and inducible targeting of all renal tubular compartments (proximal, distal tubules and collecting ducts). *Pax8CreER* ^*T2*^ transgenic mice were crossed with *Vhl* ^*fl/fl*^, *Pbrm1* ^*fl/fl*^ and *Vhl* ^*fl/fl*^;*Pbrm1* ^*fl/fl*^ conditional animals to generate compound mutants.

Tamoxifen administration induced deletion of target genes to generate experimental (*Vhl* ^*−/−*^, *Pbrm1* ^*−/−*^ and *Vhl* ^*−/−*^;*Pbrm1* ^*−/−*^) and control (*Vhl* ^*fl/fl*^, *Pbrm1* ^*fl/fl*^ and *Vhl* ^*fl/fl*^;*Pbrm1* ^*fl/fl*^) animals (Supplementary Fig. 3a,b). Mice were treated with MMC and their kidneys were assayed for markers of DNA damage. Post MMC treatment, kidneys from *Vhl* ^*−/−*^ animals demonstrated a threefold increase in the number of damaged tubular epithelia and induction of cell cycle arrest (Fig. 1d and Supplementary Fig 3c). Within individual animals, the extent of observed DNA damage closely correlated with the degree of VHL deletion, as assayed by CA9 positivity (Supplementary Fig. 3d). Significantly, *Vhl* ^*−/−*^;*Pbrm1* ^*−/−*^ mice did not display hypersensitivity to MMC nor γH2AX/CA9 correlation, despite displaying an equivalent extent of VHL loss to *Vhl* ^*−/−*^ cohorts (Supplementary Fig. 3d). Altogether, these data show that, both in vitro and in vivo, loss of VHL induces replication stress that is rescued upon concomitant loss of PBRM1.

### *Vhl* loss induces replication fork instability and collapse

**Fig. 2:**
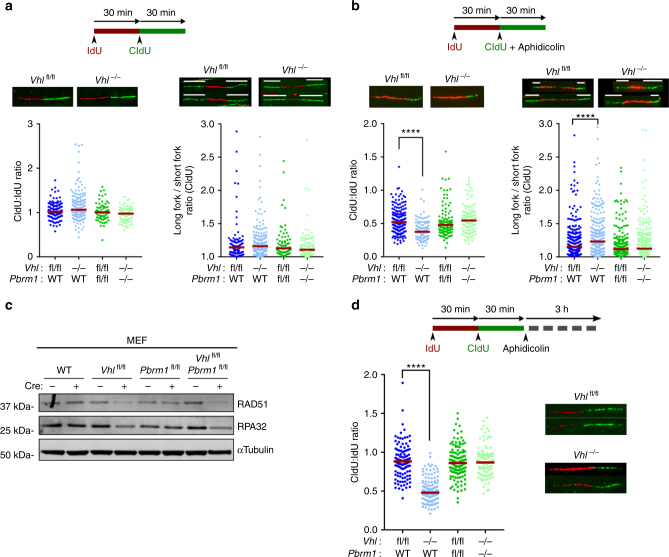
Loss of VHL induces replication fork stalling and collapse. **a**, **b** DNA fibre analyses following the indicated treatments. Top: DNA fibre assay experimental design. Bottom left: Representative images of labelled replication tracts and dot plot of CIdU:IdU track ratio in MEFs of indicated genotype (data from representative experiment; *n* > 85 tracts analysed for each genotype per experiment, *n* (independent experiments) = 3). Bottom right: Representative images of replication forks (white tracts indicate the lengths of the bidirectional forks analysed) and quantification of replication fork asymmetry (*n* > 50 forks in total per genotype, data pooled from *n* (independent experiments) = 3). **c** Representative immunoblots of MEFs. **d** Top: Experimental design of fork protection assay. Bottom left: Dot plot of of CIdU:IdU track ratio in MEFs (data from representative experiment, *n* > 220 tracts analysed for each genotype per experiment, *n* (independent experiments) = 3). Bottom right: Representative images of labelled replication tracts. **a**, **b**, **d** Median values shown in red; Kruskall–Wallis, Dunnet’s correction ****p < 0.0001

To directly explore the effect of *Vhl* inactivation on DNA replication, we evaluated replication fork dynamics using DNA fibre assays. Under unperturbed conditions, we observed no differences in replication fork progression and sister fork symmetry (Fig. 2a and Supplementary Fig. 4a). However, upon treatment with aphidicolin, *Vhl* ^*−/−*^ MEFs showed impaired fork progression and evidence of sister fork asymmetry consistent with fork stalling (Fig. 2b and Supplementary Fig. 4b), implicating VHL in the maintenance of DNA synthesis under conditions of replicative stress.

Following on from these observations we sought to determine how the loss of VHL may impact on DNA replication. The best studied function of VHL is its role in cellular oxygen sensing^3^. Loss of VHL results in activation of cellular responses to hypoxia despite normal oxygen conditions. Hypoxia has been implicated in replication stress and the potentiation of genomic instability through transcriptional and/or translational down regulation of DNA repair pathways^30–32^. We, therefore, hypothesised that the pseudohypoxia induced by VHL loss might have a similar effect. Indeed, VHL depleted MEFs showed a significant reduction in the levels of RAD51 and replication protein A (RPA32), two essential regulators of the replication stress response^33, 34^ (Fig. 2c and Supplementary Fig. 4c). We observed no change in RAD51 and RPA32 transcript levels suggesting that transcriptional repression does not significantly account for the observed depletion of RAD51 and RPA upon loss of VHL (Supplementary Fig. 4d). As both RAD51 and RPA have essential roles in preventing the degradation of stalled forks and ensuring protection of nascent DNA^35, 36^ we used DNA fibre assays to investigate fork stability in the context of *Vhl* deletion. Nascent replication tracts were IdU and CIdU-labelled before treatment with aphidicolin and replication fork stalling. Label retention (CIdU:IdU ratio) was assessed as a measure of fork stability. *Vhl* ^*−/−*^ MEFs displayed a significant reduction of median CIdU:IdU tract ratios (Fig. 2d and Supplementary Fig. 4e), indicating that VHL loss induces fork instability and collapse, an effect consistent with the observed depletion of RAD51 and RPA cellular pools.

### PBRM1 loss induces H3K9me3-marked chromatin reorganisation

**Fig. 3:**
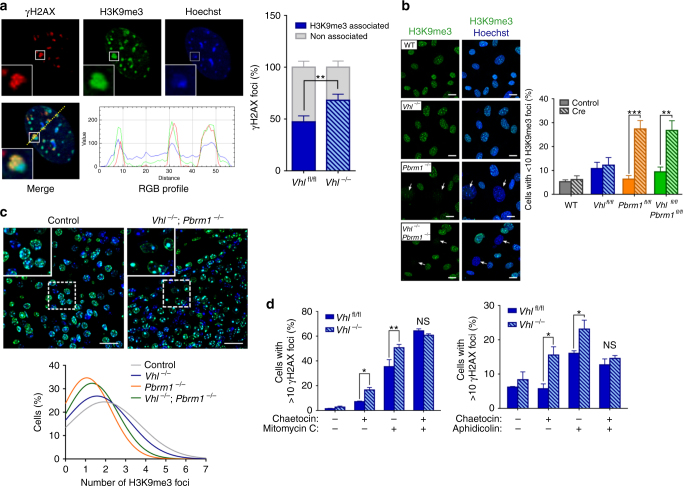
PBRM1 loss reorganises H3K9me3-marked heterochromatin to reverse VHL-induced replication stress. **a** Left: representative images of immunofluorescence staining of γH2AX and H3K9me3 in *Vhl-*deleted MEFs (*Vhl* ^*−/−*^) at 24 h after Mitomycin C. Line scans of γH2AX, H3K9me3 and Hoechst signal intensity are presented in the panels below. Right: quantification of γH2AX and H3K9me3 foci association (*n* (independent experiments) = 3). **b** Left: representative images of H3K9me3 immunofluorescence in day 8 MEFs. Arrows indicate cells with indistinct H3K9me3 foci. Scale bars, 20 μm. Right: Quantification of H3K9me3 foci (*n* (independent experiments) = 5). **c** H3K9me3 immunofluorescence in the kidneys of control and mutant mice. Representative images are shown in the top panels and histogram distributions of the number of H3K9me3 foci per cell are shown in the bottom (*n* = 4 mice per genotype; at least 6000 cells analysed per animal). Scale bars, 100 μm. **d** Quantification of γH2AX foci formation at 24 h following treatment of MEFs as indicated (*n* (independent experiments) = 3). Graphs depict mean ± s.e.m. (error bars). (**a**, **d**) Two-tailed paired *t*-test. (**b**) Two-way ANOVA, Sidak’s correction. ***P < 0.001; **P < 0.01; *P < 0.05; NS not significant

Though RAD51 and RPA remain significantly reduced in double-mutant cells, these cells display no replication fork defects (Fig. 2b, d). Furthermore, we did not observe any significant differences in the activation of DNA damage responses in PBRM1 depleted cells (Supplementary Fig. 4f), suggesting that PBRM1 loss bypasses VHL-induced replication stress through an alternative pathway. PBRM1 encodes BAF180, a component of the SWI/SNF chromatin–remodeller complex PBAF, capable of modulating DNA/chromatin interactions^37^. Recent studies have shown that the chromatin context in which replication stress occurs can strongly influence DNA damage responses, with the preferential enrichment of γH2AX in heterochromatic regions marked by H3K9me3^31, 38, 39^. Furthermore, the modulation of chromatin structure and specifically H3K9me3 is able to modify DNA replication, restore repair capacity and suppress DNA damage^38–40^. We, therefore, ascertained the chromatin localisation of DNA damage observed following MMC in *Vhl* ^*−/−*^ MEFs and found that γH2AX foci were preferentially associated with H3K9me3-marked chromatin (Fig. 3a). We thus hypothesised that PBRM1 loss could alleviate replication stress by modifying chromatin structure.

We first examined the distribution of nuclear H3K9me3 in MEFs. Although we detected no change in the total levels of cellular H3K9me3 between different genotypes, there was a striking difference in its distribution, with *Pbrm1* ^*−/−*^ and *Vhl* ^*−/−*^;*Pbrm1* ^*−/−*^ MEFs displaying more diffuse staining patterns and a significant reduction in the number of distinct foci (Fig. 3b and Supplementary Fig. 5a). Furthermore, examination of H3K9me3 in the kidneys of control, *Vhl* ^*−/−*^, *Pbrm1* ^*−/−*^ and *Vhl* ^*−/−*^;*Pbrm1* ^*−/−*^ mice revealed a similar shift in distribution patterns, with an overall reduction in the number of discernible foci in mice lacking PBRM1 (Fig. 3c and Supplementary Fig. 5b). The effect of PBRM1 loss on chromatin structure appeared to be specific to H3K9me3 with no differences observed in other heterochromatin markers (Supplementary Fig. 5c,d).

To ascertain whether H3K9me3 modulation is able to rescue VHL-dependent replication stress, *Vhl* ^*−/−*^ MEFs were treated with the histone lysine methyltransferase inhibitor chaetocin^41^ at doses sufficient to reduce the number of distinct H3K9me3 nuclear foci without depleting modification levels (to recapitulate loss of PBRM1; Supplementary Fig. 5e–g). Treatment with chaetocin was able to reverse the previously observed, VHL-induced MMC and APH hypersensitivity (Fig. 3d) suggesting that the rescue of VHL-induced replication stress by PBRM1 is mediated at least in part by its effects on H3K9me3 organisation.

### PBRM1 loss restores the cellular fitness of VHL-deleted cells

**Fig. 4:**
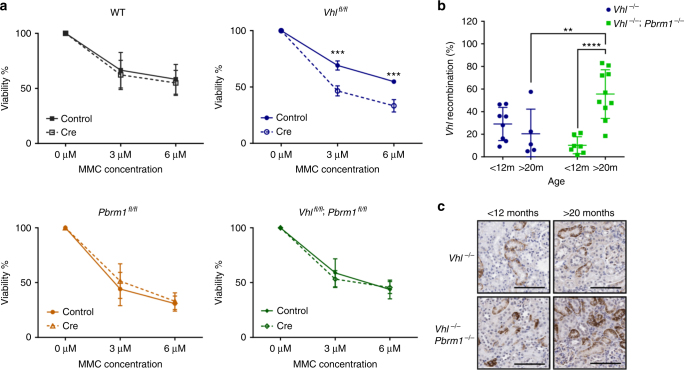
Combined loss of VHL and PBRM1 improves cellular fitness. **a** MEF viability at 6 days following exposure to Mitomycin C (*n* (independent experiments) = 4). **b** qPCR analysis of the recombined *Vhl* allele present in renal cortical cells of tamoxifen treated *Vhl* ^fl/fl^ (*Vhl* ^*−/−*^) and *Vhl* ^fl/fl^;*Pbrm1* ^fl/fl^ (*Vhl* ^*−/−*^;*Pbrm1* ^*−/−*^), (*Vhl* ^*−/−*^: *n* = 8 mice <12 m; 5 mice >20 m. *Vhl* ^*−/−*^;*Pbrm1* ^*−/−*^: *n* = 7 < 12 mice, 10 mice >20 m). **c** Representative images of CA9 immunohistochemistry of *Vhl* ^*−/−*^ and *Vhl* ^*−/−*^;*Pbrm1* ^*−/−*^ mice at indicated ages. Graphs depict mean ± s.e.m. (error bars). (**a**) Two-way ANOVA, Sidak’s correction. (**b**) Two-way ANOVA, Tukey’s correction. ****P < 0.0001; ***P < 0.001; **P < 0.01

Replication stress and the ensuing DNA damage induce checkpoint activation and cell cycle arrest, thereby curtailing cellular proliferation and cancer growth^42^. We therefore hypothesised that loss of *PBRM1* and subsequent rescue of the DNA damage caused by *VHL* deletion will confer a cellular fitness advantage to these cells. Though primary *Vhl* ^*−/−*^ and *Vhl* ^*−/−*^;*Pbrm1* ^*−/−*^ MEFs showed no difference in their proliferation rates under unperturbed culture conditions, we observed a differential response following treatment with MMC, with the reduced viability of *Vhl* ^*−/−*^ cells but not the double mutants (Fig. 4a).

The inducible nature of our genetically modified mouse models allows induction of genetic changes upon a defined time point (tamoxifen administration) thereby permitting observation and fate determination of recombined cells over time. To assess in vivo proliferative differences we quantified the allelic frequency of the recombined *Vhl* allele (*Vhl* ^*−*^) in young (<12 months) and aged (>20 months) *Vhl* ^*−/−*^ and *Vhl* ^*−/−*^;*Pbrm1* ^*−/−*^ mutant mice. Renal *Vhl* ^*−*^ allelic content did not vary with age in *Vhl* ^*−/−*^ mice (Fig. 4b). In contrast, *Vhl* ^*−/−*^;*Pbrm1* ^*−/−*^ mice showed a 5-fold increase in *Vhl* ^*−*^ allelic content with increasing age, indicating that double-mutant epithelia gain a significant proliferative advantage over *Vhl*-deleted cells. The expansion of double-mutant epithelial cells was further confirmed by CA9 immunohistochemistry (a marker of VHL loss) demonstrating a clear increase in the number of positively staining double-mutant epithelia over time (Fig. 4c). No such change was observed in *Vhl*-deleted animals.

These observations support our hypothesis that loss of PBRM1 improves the cellular fitness of VHL-defective cells permitting cellular growth and enhancing their proliferative capacity.

### *Vhl*^*−/−*^;*Pbrm1*^*−/−*^ mice develop clear cell RCCs

To ascertain whether biallelic deletion of *Vhl* and *Pbrm1* is indeed sufficient to promote renal transformation, *Vhl* ^*−/−*^, *Pbrm1* ^*−/−*^ and *Vhl* ^*−/−*^;*Pbrm1* ^*−/−*^ mice as well as control animals were aged and observed for tumour development.

**Fig. 5:**
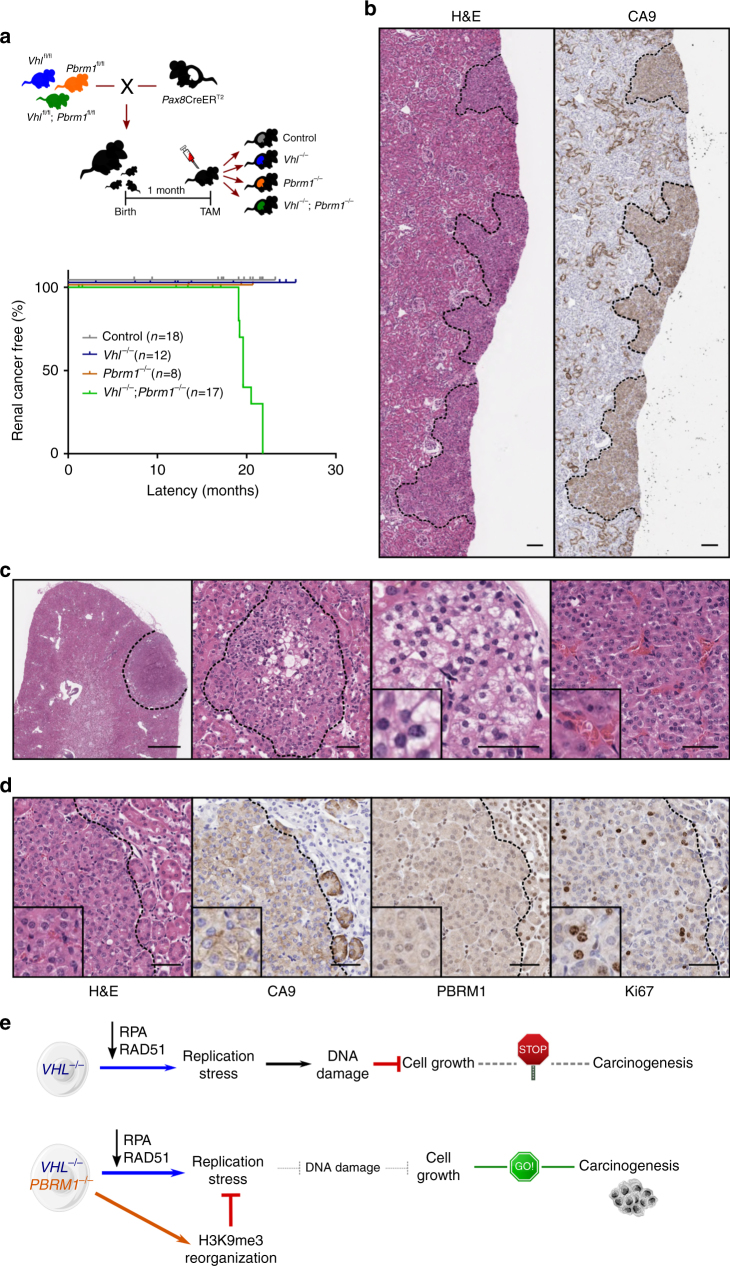
Combined loss of *Vhl* and *Pbrm1* is sufficient to initiate renal carcinogenesis. **a** Top: schematic of crossings, tamoxifen (TAM) administration and resulting cohorts. Bottom: Kaplan–Meier renal cancer-free survival of control and mutant mice. **b** Representative Haematoxylin and Eosin (H&E) and CA9 immunohistochemistry (IHC) images of serial renal sections from a *Vhl* ^*−/−*^;*Pbrm1* ^*−/−*^ mouse. Scale bars, 100 μm. **c** Representative H&E sections of renal neoplasias from *Vhl* ^*−/−*^;*Pbrm1* ^*−/−*^ mice demonstrating cytoplasmic clearing (middle panels) and prominent thin-walled vasculature (right panel). Scale bar in left panel, 1 mm. Other scale bars, 50 μm. **d** Images of serial sections of a representative renal tumour from a *Vhl* ^*−/−*^ *;Pbrm1* ^*−/−*^ mouse stained with H&E or following immunohistochemical analyses with the indicated antibodies. Scale bars, 50 μm. **e** Schematic interpretation of the effects of VHL loss alone or in combination with PBRM1 and their contributions to renal transformation

No renal tumours were observed in any of the aged control, *Vhl* ^*−/−*^ or *Pbrm1* ^*−/−*^ animals. In contrast, 100% of *Vhl* ^*−/−*^;*Pbrm1* ^*−/−*^ mice developed renal cancers by 20 months of age (Fig. 5a and Supplementary Fig. 6a). Double-mutant mice displayed a spectrum of premalignant and malignant lesions ranging from simple, benign cysts (with one to two cell layers of bland epithelium), to atypical cysts (demonstrating epithelial hyperplasia with or without mild cytological atypia) and multifocal neoplastic tumours (Fig. 5b and Supplementary Fig. 6b). Importantly, lesions arose within microscopically normal renal cortical parenchyma as is commonly observed in the sporadic cases of human ccRCC. No macroscopic metastases were observed in any of the mice. All renal lesions displayed carbonic anhydrase 9 (CA9) positivity, a diagnostic marker of human clear cell RCC and VHL inactivation^43^. *Vhl* and *Pbrm1* were confirmed to be homozygously deleted both by PCR and immunohistochemical analysis of neoplastic lesions (Fig. 5c and Supplementary Fig. 6c), indicating that these arose from double-mutant epithelia. The *Vhl* ^*−/−*^;*Pbrm1* ^*−/−*^ tumours displayed solid growth patterns with prominent vasculature and were composed of cells with eosinophilic or clear cytoplasm, and increased proliferative index, features reminiscent of the histopathological appearances of human ccRCC (Fig. 5d, e and Supplementary Fig. 6d). Renal tubular marker immunohistochemistry (megalin, Tamm–Horsfall protein (THP) and aquaporin 2, for proximal, distal tubules and collecting ducts, respectively) revealed all lesions staining strongly positive for megalin but no other renal markers, suggesting they originated from epithelia of proximal renal tubules (Supplementary Fig. 6e). Altogether, these data indicate that the combined loss of *Vhl* and *Pbrm1* within mouse tubular epithelia is sufficient for the development of renal neoplasias closely resembling the human disease.

## Discussion

Here we report the generation of a novel genetically and clinically relevant mouse model of sporadic renal cell carcinoma and provide for the first time mechanistic data shedding light into the early events driving renal carcinogenesis and the essential tumour suppressor role of *PBRM1*. Our data shows that loss of VHL induces replication stress and DNA damage accumulation, responses that curtail cellular proliferation and transformation. Significantly, we find that loss of PBRM1, through modulation of H3K9me3-marked heterochromatin, rescues VHL-dependent replication stress and confers a survival and proliferative advantage, thereby permitting renal carcinogenesis (Fig. 5f).

Replication stress is a complex phenomenon that has serious implications for genome stability, cell survival and human disease. It has being increasingly recognised as a hallmark of precancerous and early neoplastic lesions that leads to genomic instability and activation of DDR responses leading to apoptosis or senescence, thereby curtailing their growth^26^. In ccRCC replication stress has been observed in the absence of *SETD2* and is shown to enhance genomic instability and heterogeneity, impacting on cancer progression^44, 45^. Our data now establish replication stress as an early, underlying defect in the vast majority of ccRCCs, driven by the loss of *VHL* and its effects on replication fork progression and stability. Both the observed depletion of cellular RAD51 and RPA levels, two proteins essential for the protection and restart of replication forks^33, 34^, as well as the induction of replication stress have been previously associated with conditions of hypoxia^30, 31^, suggesting that these are likely to represent HIF-dependent responses. Importantly, our results provide a biological explanation for the lack of transformation upon loss of VHL alone and the need for additional genetic hits to initiate renal carcinogenesis.

To overcome oncogene-induced replication stress and allow tumour progression, a second, essential step in cancer development involves suppression of the DDR responses, commonly achieved through mutations targeting *TP53*, *ATM* or *MDM2* genes^26^. Mutations of these genes though commonly observed in other tumour types are rare in ccRCC^46^. Our data shows that in the case of ccRCC, rescue of the replication stress response can be achieved through loss of the SWI/SNF chromatin remodeller PBRM1 and appears to be mediated, at least in part, through changes in H3K9me3-marked heterochromatin. H3K9me3 has been previously shown to play a critical role in linking chromatin structure to replication and the DDR response^31, 40, 47^. Furthermore, modulation of H3K9me3 can rescue the DNA repair defects observed with ATM deficiency^38^ as well as the damage accumulation and early senescence associated with progeria^39^. The mechanisms underlying these observations are currently unclear but are thought to involve modulation of DDR responses. Therefore, one mechanism by which loss of heterochromatin organisation (in the absence of PBRM1) could rescue the VHL-induced replication defects is through alterations in DDR signalling. Though we see no evidence of a significant defect in the activation of DNA damage responses upon PBRM1 loss, further detailed investigation in future studies is required to precisely define the molecular interactions underlying these findings. Whatever the precise mechanism of replication stress rescue, both our in vitro and in vivo data clearly show that loss of PBRM1 restores the cellular fitness and proliferative potential of *Vhl*-deleted cells and is therefore critical for renal transformation.

*SWI/SNF* genes are commonly mutated across multiple cancer types and recent studies have reported their role in controlling chromatin structure and subsequent gene expression for the suppression of tumour development^48–50^. Our findings identify additional tumour suppressive functions mediated by SWI/SNF genes beyond the control of gene expression and highlight the interaction of chromatin structure and replication stress in initiating carcinogenesis. As a number of histone modifiers have been implicated in ccRCC development it will be of great interest to establish their precise interactions with VHL loss and the ensuing replication stress response.

Finally, we report the development of a novel genetically and clinically relevant mouse model of ccRCC representative of the sporadic form of the disease. While this manuscript was under preparation two groups reported the generation of mice with targeted disruption of *Vhl* and *Pbrm1* in the kidney^51, 52^. Nargund et al.^51^ induced *Vhl* and *Pbrm1* deletion using a traditional Ksp-Cre driver with mice developing renal hydronephrosis, polycystic kidneys, renal failure and occasional renal tumours (observed in 30% of mice). In the second study, Gu et al.^52^ utilised the same promoter as used here (Pax8) to drive constitutive expression of Cre recombinase from early kidney development, with mice developing renal cancers of similar histological appearances to the ones observed in our model. In contrast to these studies, our use of *Pax8*-*CreER* ^*T2*^ transgene allows for temporal and spatial control of *Vhl* and *Pbrm1* deletion and, therefore, the specific targeting of fully developed renal tubular epithelium. Thereby our study provides proof that ccRCC can arise from the fully differentiated epithelium of renal proximal tubules. Furthermore, the inducible nature of our model avoids potential confounding by effects of *Vhl* and *Pbrm1* deletion during renal embryogenesis (as observed in the other RCC mouse models). As a consequence, the tumours observed develop within normal surrounding renal parenchyma and without signs of morbidity, representing an accurate model of early, sporadic human ccRCC and therefore a useful tool for the discovery and preclinical evaluation of new methodologies for early detection, prevention and treatment of the disease. Importantly, our study provides essential insights into the molecular mechanisms underpinning renal carcinogenesis, highlighting replication stress as an underlying vulnerability for the majority of ccRCC and providing opportunities for the development of much needed novel therapeutics.

## Methods

### Data reporting

No statistical methods were used to predetermine sample size. The experiments were not randomised and the investigators were not blinded to allocation during experiments. For outcome assessment most experiments were automatically analysed by Fiji scripts to minimise potential human bias. When manual annotation was required, blinding precautions were made.

### Mouse strains and treatments

*Pax8CreER* ^*T2*^ transgenic mice have been previously described^16^. Conditional *Vhl* ^*fl/fl*^ mice were obtained from Jackson Laboratories (B6.129S4(C)-*Vhl* ^*tm1Jae*^/J)^53^. *Pbrm1* ^*fl/fl*^ mice were generated by the Wellcome Trust Sanger Institute (Hinxton, UK), using European Conditional Mouse Mutagenesis Program (EUCOMM) targeted embryonic stem cells (Pbrm1^tm1a(EUCOMM)Wtsi^; clone ID EPD0089_3_C01). Briefly, a knockout-first conditional allele composed of an *IRES:lacZ* trapping cassette and a floxed promoter-driven neomycin cassette flanking exon 4 of the *Pbrm1* gene (Extended Data Fig. 3a), was targeted in C57BL/6 N embryonic stem cells^54^. The EPD0089_3_C01 clone was passaged twice in 2i/LIF media^55^ at low density to enrich for ground state pluripotent cells. ES cell clones were isolated and expanded for injection into 8-cell embryos to produce chimeric founders. Genotyped progeny that were positive for germline transmission were bred, and first generation offspring that inherited the targeted allele were subsequently mated with FLPeR mice^56^ to remove the lacZ/neomycin selection cassette, leaving *loxP* sites on either side of exon 4 (exon 2 of the translated transcript). The FLPeR allele was bred out of the mouse line following cassette elimination. All mice were maintained on a C57BL/6 background. Mice were crossbred to produce experimental cohorts (*Pax8CreER* ^*T2*^;*Vhl* ^*fl/fl*^, *Pax8CreER* ^*T2*^;*Pbrm1* ^*fl/fl*^ and *Pax8CreER* ^*T2*^;*Vhl* ^*fl/fl*^;*Pbrm1* ^*fl/fl*^) and littermate controls (*Vhl* ^*fl/fl*^, *Pbrm1* ^*fl/fl*^ and *Vhl* ^*fl/fl*^;*Pbrm1* ^*fl/fl*^). Mouse genotypes from ear biopsies were determined using real-time PCR with specific probes designed for each gene (Transnetyx, Cordova, TN). To induce deletion of target genes 4-week to 6-week-old experimental and control animals were injected intraperitoneally with 2 mg of tamoxifen (Sigma, UK) for 5 consecutive days^16^.

For the mitomycin C (MMC) sensitivity studies, cohorts of mice 2–6 months post tamoxifen induction (3–8 months of age) were treated with either saline or MMC (0.5 mg/ml; Sigma, UK) at a single intraperitoneal injection of 5 mg/kg. Mice were euthanized 10 days post treatment and kidneys were collected for further analyses. Seven out of 39 (18%) animals showed macroscopic and microscopic signs of renal cortical glomerular and tubular atrophy, a previously reported toxicity of MMC in mice^57^. Affected mice were not of particular sex, age or genotype (4/20 controls, 2/8 *Pax8CreER* ^*T2*^;*Vhl* ^*fl/fl*^ and 1/4 *Pax8CreER* ^*T2*^;*Pbrm1* ^*fl/fl*^ *)* and were excluded from further analysis.

Animal studies were conducted using age-matched littermate controls for each experiment. Both male and female mice were used. Animals were maintained under specific pathogen free conditions in accordance to Home Office UK regulations and the Animals (Scientific Procedures) Act, 1986. All experimental protocols were approved by the Animal Welfare and Ethical Review Body (AWERB) of the University of Cambridge CRUK Cambridge Institute.

### Generation and culture of MEFs

MEFs were isolated from WT, Vhl^fl/fl^, Pbrm1^fl/fl^ and Vhl^fl/fl^;Pbrm1^fl/fl^ animals at embryonic day 12.5 and aliquots were frozen at passage 3. Cells were cultured in DMEM supplemented with 10% FBS (Thermo Fisher) and 1% penicillin/streptomycin (Thermo Fisher) and maintained in conventional cell culture incubators at 5% oxygen to replicate physiological tissue conditions^20^. For *Cre*-mediated recombination of target genes, two days after thawing, MEFs were treated for two consecutive days with 1 µM of TAT-Cre Recombinase (SCR508, Merck Millipore) and 100 µM of Chloroquine (C6628, Sigma) for 5 h; control cells were treated with 100 µM of Chloroquine only. Treatment was repeated at day 15 after recombination to ensure cultures remained fully recombined. Recombination was confirmed by PCR and immunoblotting. Data were obtained from at least three independent experiments using primary MEFs derived from a minimum of three different embryos and two separate primary cultures. For proliferation assays, cells were seeded in 12-well plates at 40,000 cells/ml in triplicate wells and counted after 4 days, before re-seeding at the same density for the next passage. Cumulative population doubling was calculated as log(N_f_/N_i_)/log_2_, where N_i_ and N_f_ correspond to number of cells plated and final counts/passage, respectively.

All cells tested negative for mycoplasma contamination.

### Immunoblotting

MEFs were lysed in RIPA buffer with protease and phosphatase inhibitors and lysates were processed using standard methods. We used the following primary antibodies: VHL (sc-5575, 1:250; Santa Cruz), PBRM1 (A301-590A, 1:1000; Bethyl Laboratories), alpha-Tubulin (T5168, 1:2000; Sigma), RAD51 (sc-8349, 1:100; Santa Cruz), RPA32 (A300-244A, 1:500; Bethyl Laboratories), pATM (ab36810, 1:1000; Abcam), ATM (ab85213, 1:1000; Abcam), pDNA-PKc (ab18192, 1:1000; Abcam), DNA-PKc (ab70250, 1:1000; Abcam), pCHK1 (#2348 S, 1:500; Cell Signalling), CHK1 (#2360, 1:1000; Cell Signalling), H3K9me3 (ab8898, 1:1000; Abcam), H3K27me3 (ab6002, 1:1000; Abcam), H3K9me2 (ab8898, 1:1000; Abcam), H4K20me3 (ab9053, 1:1000; Abcam), HP1a (ab77256, 1:1000, Abcam), KAP1 (ab10484, 1:1000; Abcam) and H3 (4499, 1:2000; Cell Signalling). Secondary antibodies were conjugated to IRDye 680 or 800 (Li-Cor). Fluorescent signals were imaged using the Odyssey Infrared Imaging System (Li-Cor). Western blot band quantifications were performed with Fiji^58^. Uncropped scans of presented blots are shown in Supplementary Fig. 7.

### Hypersensitivity assays

MEFs were seeded at 40,000 cells/ml on Ibidi 8-well µSlides (IB-80826, Ibidi) and allowed to attach for 48 h before treatment. All cells were treated with either mitomycin C (MMC) or aphidicolin (APH) at day 6 post recombination. For MMC assays cells were treated with 2 µM of MMC (Sigma) for 2 h before replacing with either fresh media or media containing 200 nM of CDKi (CDK1/2 Inhibitor III, 217714, Merck Chemicals) as specified. Alternatively, cells were treated with 75 nM of Chaetocin for 24 h before adding 2 µM of MMC for 2 h. After treatment, fresh media containing 75 nM of Chaetocin was added for a further 24 h. For APH assays, aphidicolin (A4487, Sigma) was added at 200 nM with or without 200 nM of CDKi for 24 h. Alternatively, cells were treated with 75 nM of Chaetocin for 24 h before adding aphidicolin 200 nM to the media for a further 24 h.

### Immunocytochemistry

MEFs grown on Ibidi 8-well µSlides were washed with PBS and fixed in 4% paraformaldehyde for 15 min. For pATR and pCHK1 detection, cells were permeabilized by treating them with CSK buffer (20 mM Tris, 300 mM glucose, 100 mM NaCl, 3 mM MgCl2, 0.5% Triton X-100) for 12 min at room temperature followed by incubation with CSK-S buffer (10 mM Tris, 10 mM NaCl, 3 mM MgCl2, 1% Tween, 0.5% SDS) for 12 min. For other antibodies, cells were permeabilized with 0.5% Triton X-100 in PBS for 10 min. Cells were then blocked in 2.5% normal donkey serum (NDS) for 1 h and incubated with primary antibody diluted in 1% NDS overnight at 4 °C in a humid box. Cells were incubated with Alexa fluor fluorochrome (Thermo Fisher) secondary antibodies diluted in 1% NDS for 30 min at room temperature before being incubated with Hoechst 33258 (1 μg/mL, Thermo Fisher) for 5 min. The wells were washed with PBS and covered with a drop of Mounting media (Ibidi). We used the following primary antibodies: γH2AX (9718, 1:1,000; Cell Signalling and ab26350, 1:500; Abcam), 53BP1 (IHC-00001, 1:500; Bethyl Laboratories), pATR (#2853, 1:500; Cell Signalling), pCHK1 (#2348S, 1:50, Cell Signalling), H3K9me3 (ab8898, 1:1000; Abcam), H2AZ (ab4174, 1:100; Abcam), H3K27me3 (ab6002, 1:500; Abcam), H3K9me2 (ab8898, 1:200; Abcam), H4K20me3 (ab9053, 1:100; Abcam), HP1a (ab77256, 1:200; Abcam) and KAP1 (ab10484, 1:500; Abcam). Image stacks of whole cells were obtained by confocal laser-scanning microscopy (Leica TCS SP5) and automatic counting of foci and nuclei detection was performed by an appropriate macro in Fiji. Association of γH2AX with H3K9me3 foci was analysed using Imaris software (Bitplane). In all studies more than 100 cells were analysed per genotype for each experiment.

### β-galactosidase staining

Cells were seeded at a density of 80,000 cells/ml in 12-well plate 2 days prior the staining for β-galactosidase. At day 20 after recombination cells were washed with PBS and β-galactosidase activity was assessed using the β-galactosidase staining kit (9860, Cell signalling) following the manufacture’s protocol. Images were taken with NIKON Eclipse TS100 microscope and processed with Fiji software for analysis. More than 150 cells were evaluated per genotype for each experiment.

### DNA fibre assays

Asynchronous MEFs were labelled as indicated with 50 µM iododeoxyuridine (IdU, Sigma I7125) followed by 500 µM of chlorodeoxyuridine (CldU, Sigma C6891). Aphidicolin (200 nM) was added with the second pulse of CldU or immediately after labelling with IdU as indicated. Cells were collected and resuspended in PBS at a density of 1 × 10^6^ cells/mL. 2 µL of cell suspension was spotted onto a Superfrost microscope slide (Thermo Fisher) and overlaid with 7 µL of DNA lysis buffer (200 mM Tris-HCl, 50 mM EDTA, 0.5% SDS). After 10 min, the slides were tilted by 15 degrees to allow lysates to slowly move down the slide. The DNA spreads were air-dried for 4 h, fixed in a 3:1 mixture of methanol/acetic acid for 10 min at room temperature and air-dried overnight. The slides were then treated with 2.5 N HCl for 60 min at 37 °C, washed in PBS and incubated in blocking buffer (5% BSA/PBS) for 30 min at room temperature followed by incubation with a mouse anti-BrdU antibody (to detect IdU) (347590, 1:50, BD Biosciences) and a rat anti-BrdU antibody (to detect CldU) (ab6326, 1:200, Abcam). After washing three times with PBS, the slides were incubated for 12 min in stringency buffer (36 mM Tris-HCl, 0.5 mM NaCl and 0.5% Tween), rinsed three times in PBS and incubated for 30 min with Alexa Fluor 488-conjugated anti-rat and Alexa Fluor 555-conjugate anti-mouse, washed in PBS and mounted in Mounting media (Ibidi). Fibre images were obtained in a Leica DMI 6000B microscope using a 63x objective. In each assay >100 fibres were measured and each individual experiment was repeated at least two times.

### Real-time PCR

Total RNA was isolated from MEFs using the RNeasy Mini kit (Qiagen). Purified RNA was reverse transcribed using the SuperScript II Reverse Transcriptase kit (Thermo Fisher). *Rad51* and *RPA32* levels were measured using the TaqMan gene expression assays and a Quantstudio 6 Instrument (Applied Biosystems). Real-time quantitative PCRs were carried out following the manufacture’s protocol using the following probes: Mm00488047_m1 (*RPA2*), Mm00487905_m1 (*Rad51*), Mm00437762_m1 (*B2M*), Mm03024075_m1 (*HPRT*). Relative sample quantification was carried out by the comparative DDCt method using B2M and HPRT as endogenous controls.

### Viability assays

At day 4 after recombination MEFs were seeded at 20,000 cells/ml in triplicate in 96-well plates and allowed to attach for 48 h before treatment. Mitomycin C was added to cells for 2 h at the stated concentrations before medium was replaced with fresh growth medium. Viability was analysed 6 days following treatment using MTT assay (CGD1, Sigma) according to the manufacturer’s protocol.

### Histology and immunohistochemistry

At experimental end-points animals were euthanized and tissues were fixed in 4% paraformaldehyde in PBS overnight and embedded in paraffin. To review histology, 4 µm sections were stained with haematoxylin and eosin (H&E). Immunohistochemistry (IHC) and immunofluorescence (IF) were performed on 4 µm tissue sections using standard protocols. Specificity of immunostaining was assessed by incubation in the absence of primary or secondary antibody. We used the following primary antibodies: γH2AX (9718, 1:1000; Cell Signalling), H3K9me3 (ab8898, 1:500; Abcam), p21 (sc-471, 1:2000; Santa Cruz), ki67 (NCL-ki67p, 1:1000; Novacastra), PBRM1 (A301-591A, 1:2000; Bethyl Laboratories), CA9 (sc-25600, 1:200; Santa Cruz), aquaporin 2 (ab105171, 1:1000; Abcam), megalin (ab76969, 1:1000; Abcam) and Tamm–Horsfall protein (THP; AF5175, 1:1000; R&D Systems). Secondary antibodies used were conjugated to HRP (IHC) or Alexa fluor fluorochromes (IF). Fluorescent images were obtained by confocal laser-scanning microscopy (Leica TCS SP5). All histology was independently reviewed by a specialist renal pathologist.

For quantification of γH2AX, p21 and ki67 expression, stained tissue sections were scanned with the Aperio ScanScope (Aperio, Vista, CA) and images were visualised and captured using the Aperio ImageScope program. Quantification was performed on a minimum of 15 fields (×20 magnification) from three representative coronal kidney sections per animal. Images were processed using Fiji software by calculating the percentage of DAB positive nuclei relative to total number of nuclei within the field (a minimum of 8000 cells were analysed per animal).

For quantification of H3K9me3, a minimum of 10 image stacks were obtained from at least 2 coronal sections of kidney per animal by confocal laser-scanning microscopy (Leica TCS SP5). Nuclei detection and foci counting was performed automatically by an appropriate macro in FIJI. At least 6000 cells were analysed per animal. For quantification of γH2AX/CA9 co-immunostaining, a minimum of six image stacks were obtained from at least two coronal sections of kidney per animal by confocal laser-scanning microscopy (Leica TCS SP5). Staining was quantified automatically by an appropriate macro in FIJI. At least 10,000 cells were analysed per animal.

### Quantitative PCR

Genomic DNA from macroscopically normal renal cortical parenchyma was extracted using standard protocols. For quantitative PCR (qPCR) of the *Vhl* deletion, SYBR Green PCR Master Mix (Applied Biosystems) was used and amplification and analysis was carried out on QuantStudio 6 (Thermo Fisher Scientific). Primers forward 5′-CTGGTACCCACGAAAGTGTC-3′ and reverse 5′-CTGACTTCCACTGATGCTTGTCACAG-3′ were used to detect the recombined allele (*Vhl* ^*−*^). Serial dilutions of fully recombined *Vhl* ^*−/−*^ MEF DNA were used to construct a standard curve and to determine *Vhl* ^*−*^ allelic frequency.

### Genotyping

Genotyping of MEFs and additional genotyping of mice was performed by PCR using the following primers and conditions. *Vhl* allele recombination: forward primer for floxed allele 5′-CCGGAGTAGGATAAGTCAGCTGAG-3′, forward primer for recombined allele 5′-CTGGTACCCACGAAAGTGTC-3′, common reverse primer 5′-CTGACTTCCACTGATGCTTGTCACAG-3′ (400 bp product for floxed allele, 200 bp product for WT and 250 bp product for recombined allele); 1 cycle of 10 min 94 °C, 55 cycles of (50 sec 95 °C, 50 sec 58 °C, 60 sec 72 °C), 1 cycle of 5 min 72 °C^16^. *Cre recombinase*: forward primer 5′-GCACTGATTTCGACCAGGTT-3′, reverse primer 5′-GCTAACCAGCGTTTTCGTTC-3′ (200 bp product); *Pbrm1* allele recombination: forward primer 5′-GCAGGAGAGTTATATAAGCCAATAAGCTG-3′, reverse primer 5′-TGACACCAAAGAGTTCTCCAGGATCA-3′ (1266 bp product for floxed allele, 1119 bp product for WT and 423 bp product for recombined allele); 1 cycle of 1 min 95 °C, 30 cycles of (15 s 95 °C, 30 s 58 °C, 1 min 68 °C), 1 cycle of 5 min 68 °C.

### Statistical analysis

Data were visualised and statistical analyses performed using Prism 7.0 software (Graph Pad). *P* < 0.05 was considered statistically significant. For cases where experimental groups showed comparable variance the following statistical tests were applied. *P*-values for unpaired comparisons between two groups were calculated by two-tailed Student’s *t*-test, whilst *P*-values for paired comparisons were calculated by two-tailed paired *t*-test. One-way analysis of variance (ANOVA) was used for analysis between three or more groups, followed by appropriate multiple comparison correction. Ordinary two-way ANOVA was used for analysis that involved two variables, followed by appropriate multiple comparison correction. For experiments showing non-comparable variance between groups the Kruskall–Wallis test was applied followed by Dunn’s correction for multiple comparisons. *P < 0.05; **P < 0.01; ***P < 0.001; ***P < 0.0001. Error bars, mean ± s.d. or s.e.m., as indicated.

### Data availability

The data that support the findings of this study are available within the Article and Supplementary Files, or available from the authors upon request.

## Electronic supplementary material

Peer Review File

Supplementary Information
