# Supplementary material for: Loss of PBRM1 rescues VHL dependent replication stress to promote renal carcinogenesis
Source: Nat Commun. 2017 Dec 11;8:2026. doi: 10.1038/s41467-017-02245-1 (PMC5725450; doi:10.1038/s41467-017-02245-1)
Supplement: Supplementary file 2 — Supplementary Information [file 41467_2017_2245_MOESM2_ESM.docx]

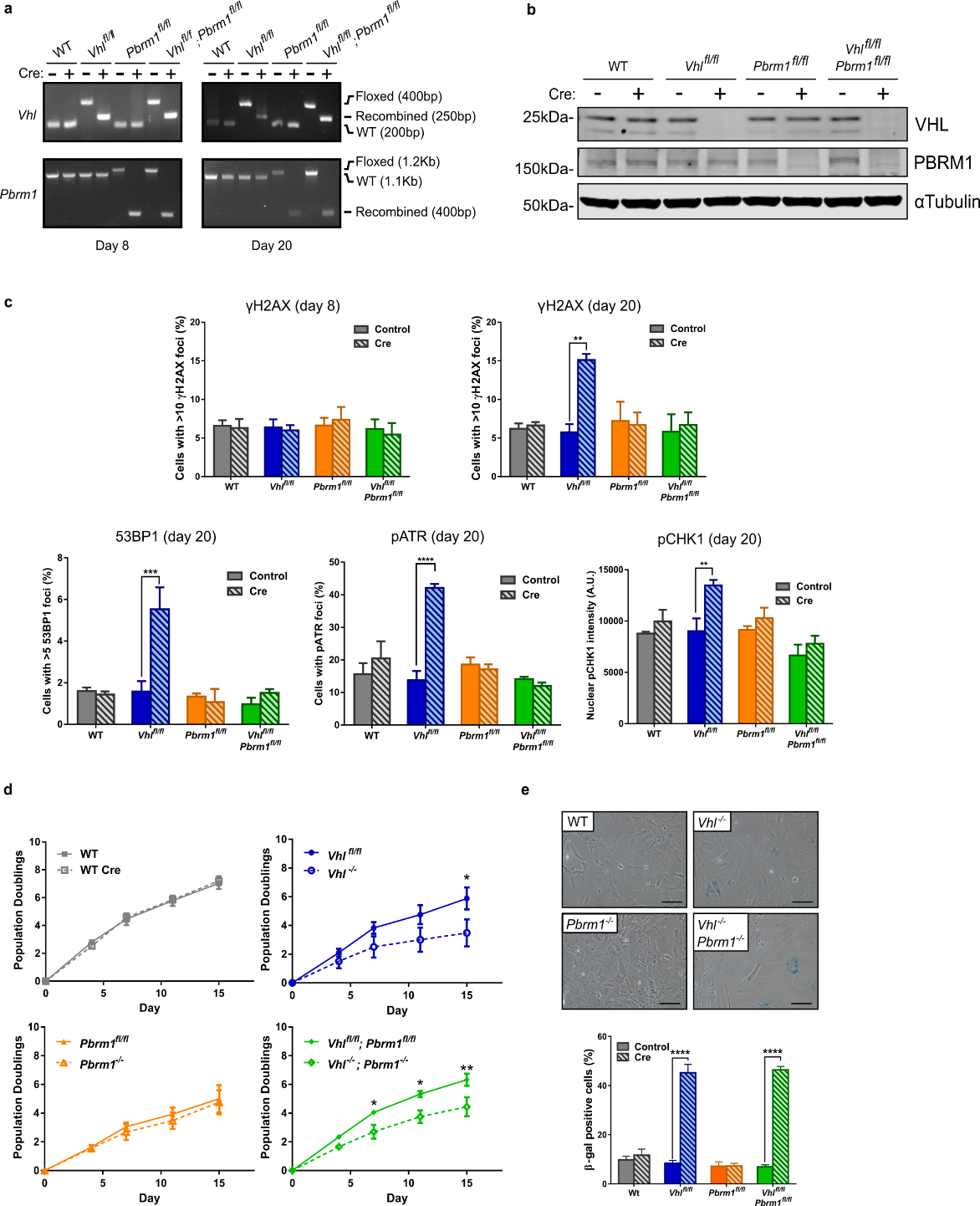


**Supplementary Figure 1. *Vhl* but not *Vhl;Pbrm1* deleted MEFs accumulate DNA damage with increasing passage. a,** Representative data of PCR analysis of wild-type (WT), *Vhl^fl/fl^*, *Pbrm1^fl/fl^* and *Vhl^fl/fl^;Pbrm1^fl/fl^* MEFs at day 8 or 20 post treatment with TAT-Cre (Cre). **b,** Representative immunoblot of MEFs 8 days post treatment. **c,** MEFs were treated with either chloroquine (control) or TAT-Cre recombinase (Cre) and the presence of γH2AX, 53BP1, pATR and pCHK1 was quantified at indicated days (γH2AX, n (independent experiments)=4; 53BP1, pATR, pCHK1 n (independent experiments)=3); A.U.: arbitrary units. **d,** Proliferation rates of MEFs with or without allelic deletion (n (independent experiments)=4). **e,** MEFs were assayed for β-galactosidase positivity at 20 days post treatment (n (independent experiments)=4). Representative images are shown in the top panels and staining quantification in the bottom. Graphs depict mean ± s.e.m. (error bars). Two-way ANOVA, Sidak’s correction. *****P*<0.0001; ****P*<0.001; ***P*<0.01; **P*<0.05. Scale bars, 100 μm.


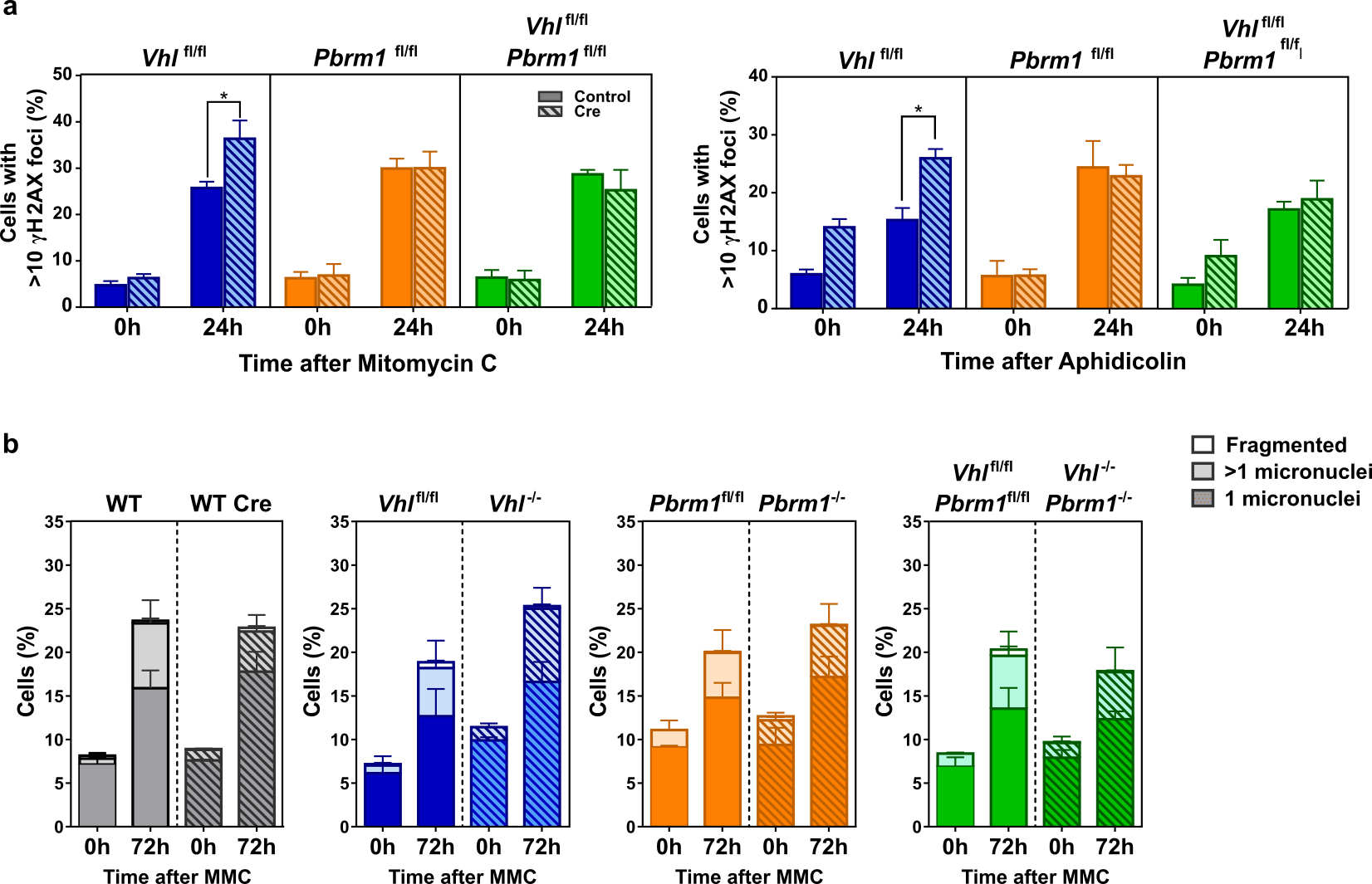


**Supplementary Figure 2. VHL depleted cells are hypersensitive to mitomycin C and aphidicolin.**

**a,** Quantification of γH2AX foci formation following treatment of MEFs as indicated (n (independent experiments)=3). **b,** Quantification of nuclear defects observed in MEFs 72 hours after treatment with MMC. (n (independent experiments)=4). Graphs depict mean ± s.e.m. (error bars). **a,** Two-way ANOVA, Sidak’s correction. **d,** Two-way ANOVA, Tukey’s correction. **P*<0.05.


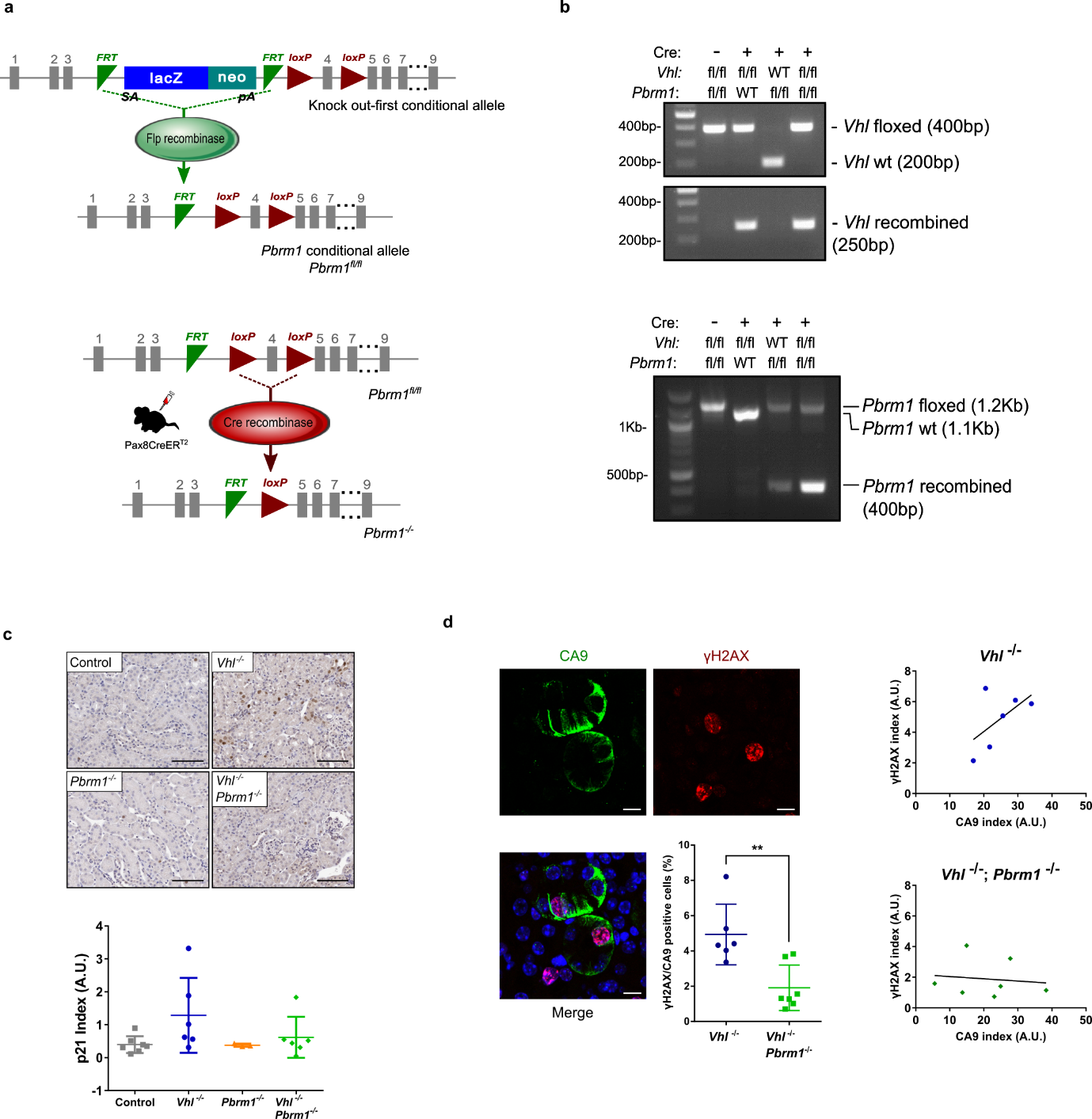


**Supplementary Figure 3. VHL loss induces Mitomycin C hypersensitivity *in-vivo*. a.** Schematic diagram of the generation of conditional *Pbrm1* deleted mice (*Pbrm1^-/-^*). SA: splice acceptor; pA: poly-adenylation signal; neo: neomycin. **b,** PCR analysis of recombination at the *Vhl* and *Pbrm1* locus in the kidneys of mice with combinations of *Pax8-CreER^T2^*, *Vhl* floxed (fl), *Pbrm1* floxed and wild-type (WT) alleles. The positions of the bands representing the floxed, WT and recombined alleles are indicated. **c,** Representative photos of p21 immunohistochemistry in Mitomycin C treated mice and p21 quantification (n (mice); Control=7, *Vhl^-/-^* =6, *Pbrm1^-/-^* =3, *Vhl^-/-^;Pbrm1^-/-^* =6). Scale bars, 100 μm. **d,** Left: Representative photos of co-immunofluorescence staining in *Vhl^-/-^* mice and quantification of double positive cells (n (mice); *Vhl^-/-^* =6, *Vhl^-/-^;Pbrm1^-/-^* =7; at least 10,000 cells analysed per animal). Scale bars, 10 μm. Right: Pearson correlation between γH2AX and CA9 index in MMC treated animals (n (mice); *Vhl^-/-^* =6, *Vhl^-/-^;Pbrm1^-/-^* =7). **c, d,** Graphs depict mean ± s.d. (error bars); A.U.: arbitrary units. **d,** Unpaired t-test. ***P*<0.01.

**
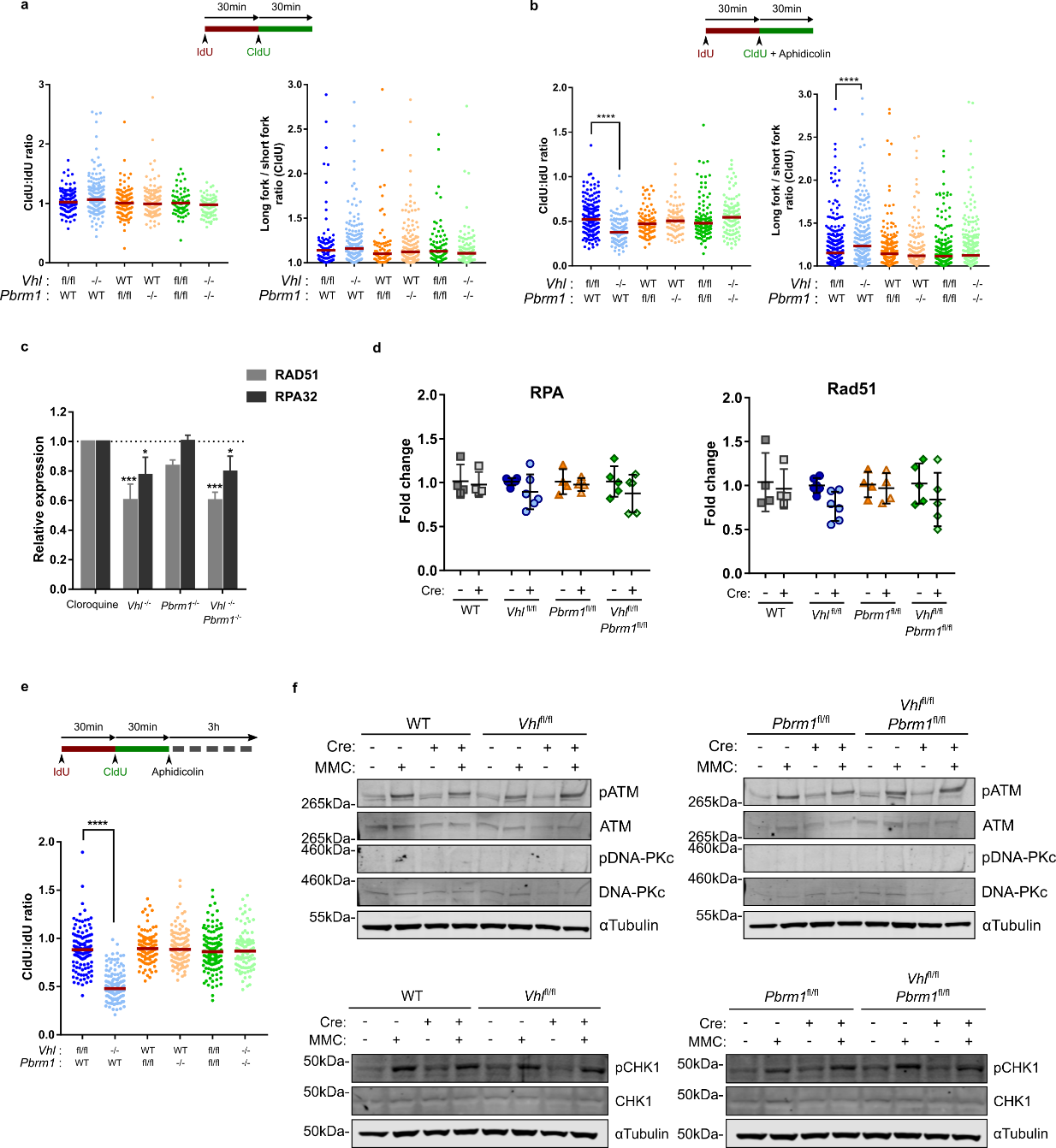
**

**Supplementary Figure 4. Loss of VHL induces replication fork stalling and instability. a, b,** Top: DNA fibre assay experimental design under **(a)** unperturbed and **(b)** replication stress conditions. CIdU:IdU track ratios in MEFs of indicated genotype (data from representative experiment; n>85 tracts analysed for each genotype per experiment, n(independent experiments)=3) and quantification of replication fork asymmetry (n>150 forks in total per genotype, data pooled from n(independent experiments)=3) for each condition, are presented in the panels below. **c,** Relative quantification of RAD51 and RPA32 protein levels of protein lysates of TAT-Cre treated MEFs compared to their chloroquine treated controls (n(independent experiments)=4). **d,** Real-time RT-PCR analysis of *Rad51* and *RPA32* mRNA in MEFs (n (independent experiments); WT=4, *Vhl^fl/fl^*=6, *Pbrm1^fl/fl^*=4, *Vhl^fl/fl^;Pbrm1^fl/fl^*=5). **e,** Top: Experimental design of fork protection assay. Bottom: Dot plot of IdU track length in MEFs (data from representative experiment; n>220 tracts analysed for each genotype per experiment, n(independent experiments)=3). **f,** Representative immunoblots of whole cell extracts from MEFs of indicated genotypes, 24 hours following treatment with mitomycin C. **a, b, e,** Median values shown in red. **a, b,** Kruskall-Wallis, Dunn’s correction. **c,** Graphs depict mean ± s.e.m. (error bars); Two-way analysis of variance (ANOVA), Dunnett’s. **d,** Graph depicts mean ± s.d. (error bars). **e,** One-way ANOVA, Sidak’s correction. *****P*<0.0001; ****P*<0.001; **P*<0.05.


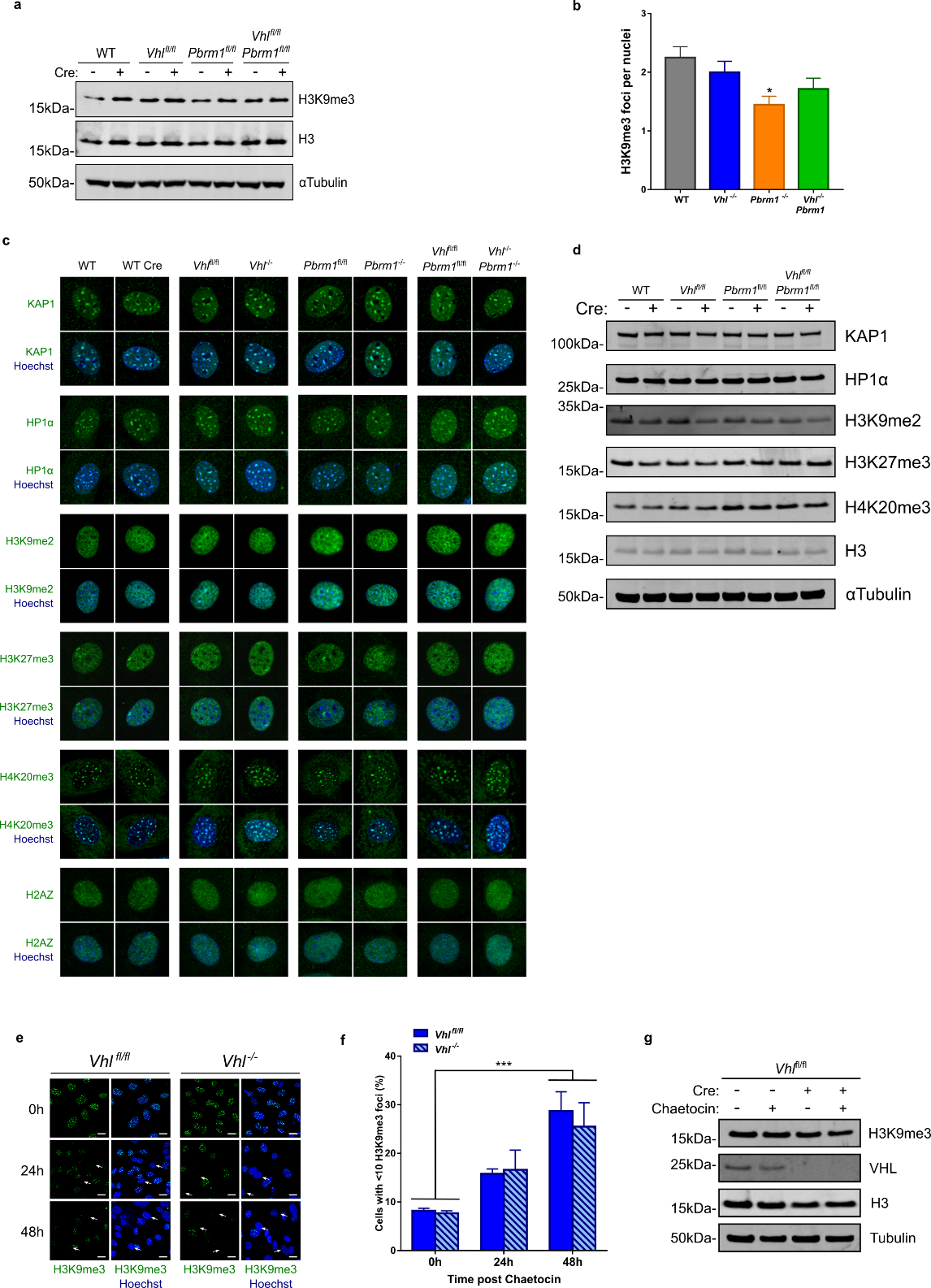


**Supplementary Figure 5. Loss of *Pbrm1* induces H3K9me3 reorganisation. a,** Representative immunoblot of MEFs 8 days post treatment. **b,** Quantification of nuclear H3K9me3 immunofluorescent foci in renal cortical cells of control and mutant mice (n=4 mice per genotype; at least 6000 cells analysed per animal). **c,** Representative immunofluorescence images of heterochromatin markers in MEFS. **d,** Representative immunoblot of MEFs 8 days post treatment. **e-g,** *Vhl* MEFs were treated with chaetocin and H3K9me3 was analysed at 24 and 48hrs. **e,** Representative immunofluorescence images (arrows indicate cells with no discernible H3K9me3 foci). **f,** Quantification of H3K9me3 foci (n (independent experiments)=6). **g,** Representative immunoblot at 48 hours post-treatment. Graphs depict mean ± s.e.m. (error bars). **b,** One-way ANOVA, Dunnett’s. **f,** Two-way ANOVA, Sidak’s correction. ****P*<0.001; **P*<0.05. Scale bar, 20 μm.


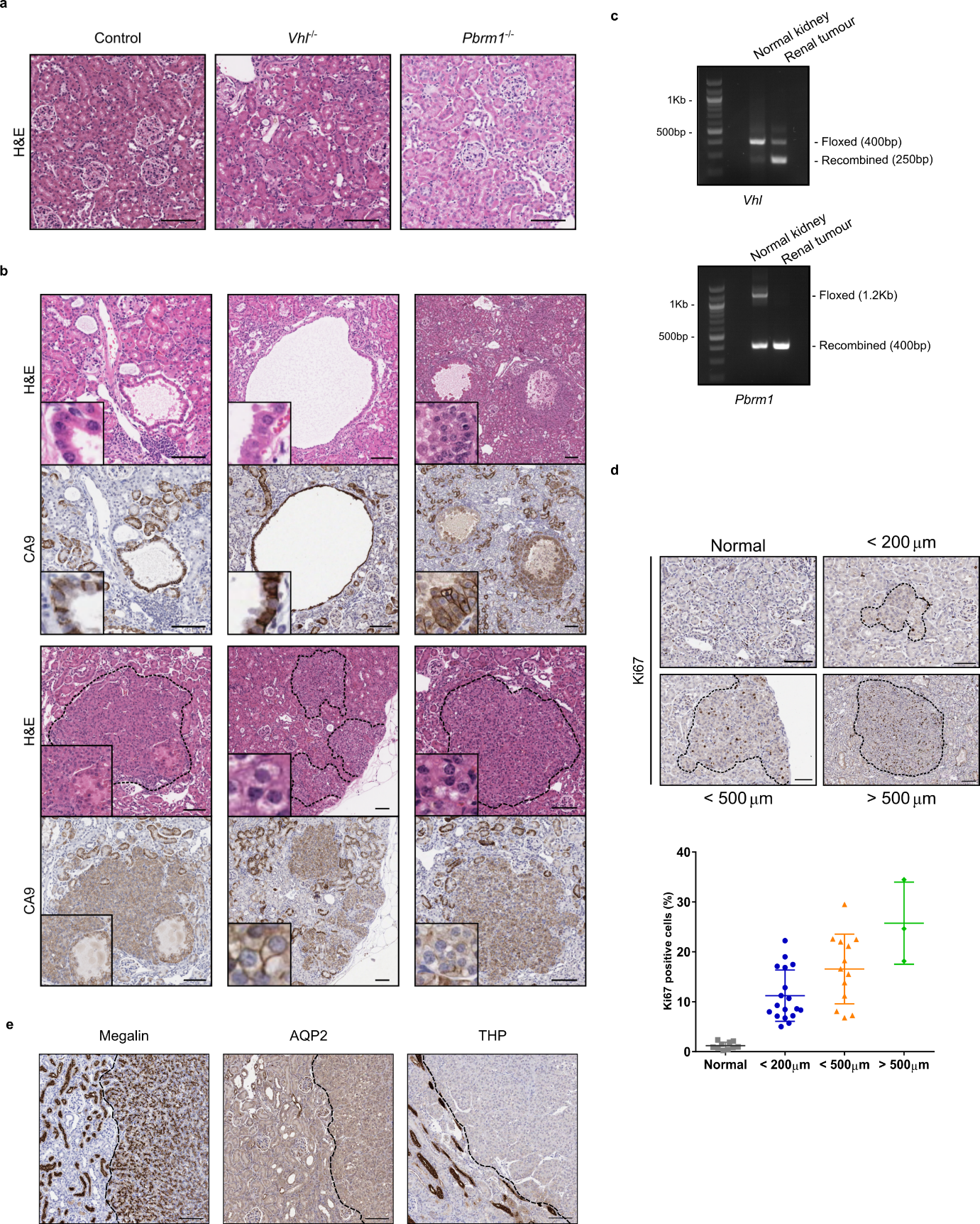


**Supplementary Figure 6. Combined loss of *Vhl* and *Pbrm1* promotes renal carcinogenesis. a,** Representative haematoxylin and eosin (H&E) renal sections from control (*Vhl^fl/fl^; Pbrm1^fl/fl^*), *Vhl^-/-^* and *Pbrm1^-/-^* mice. **b,** Representative images of H&E (top) and CA9 immunohistochemistry (bottom) of serial sections from renal lesions observed in *Vhl^-/-^;Pbrm1^-/-^* mice (top: simple and atypical/multi-layered cysts; bottom: cystic and solid neoplasias). **c,** PCR analysis of recombination at the *Vhl* and *Pbrm1* locus in normal renal parenchyma and a representative renal tumour from a double mutant mouse. The positions of the bands representing the floxed and recombined alleles are indicated. **d,** Top: representative images of ki67 immunohistochemistry in double mutant mice (neoplastic lesions are indicated). Bottom: ki67 quantification in the normal renal parenchyma and renal tumours of increasing size from *Vhl^-/-^;Pbrm1^-/-^* mice. Normal (n (mice)=10), <200µm (n (tumours)=18), <500µm (n (tumours)=13), >500µm (n (tumours)=3). Graph depicts mean ± s.d. (error bars). One-way ANOVA linear test for trend *P*<0.0001. **e.** Representative images of renal marker immunohistochemistry of a *Vhl^-/-^;Pbrm1^-/-^* renal lesion; the dashed line marks the boundary between tumour (right) and normal (left) renal parenchyma. Scale bars, 100 μm.


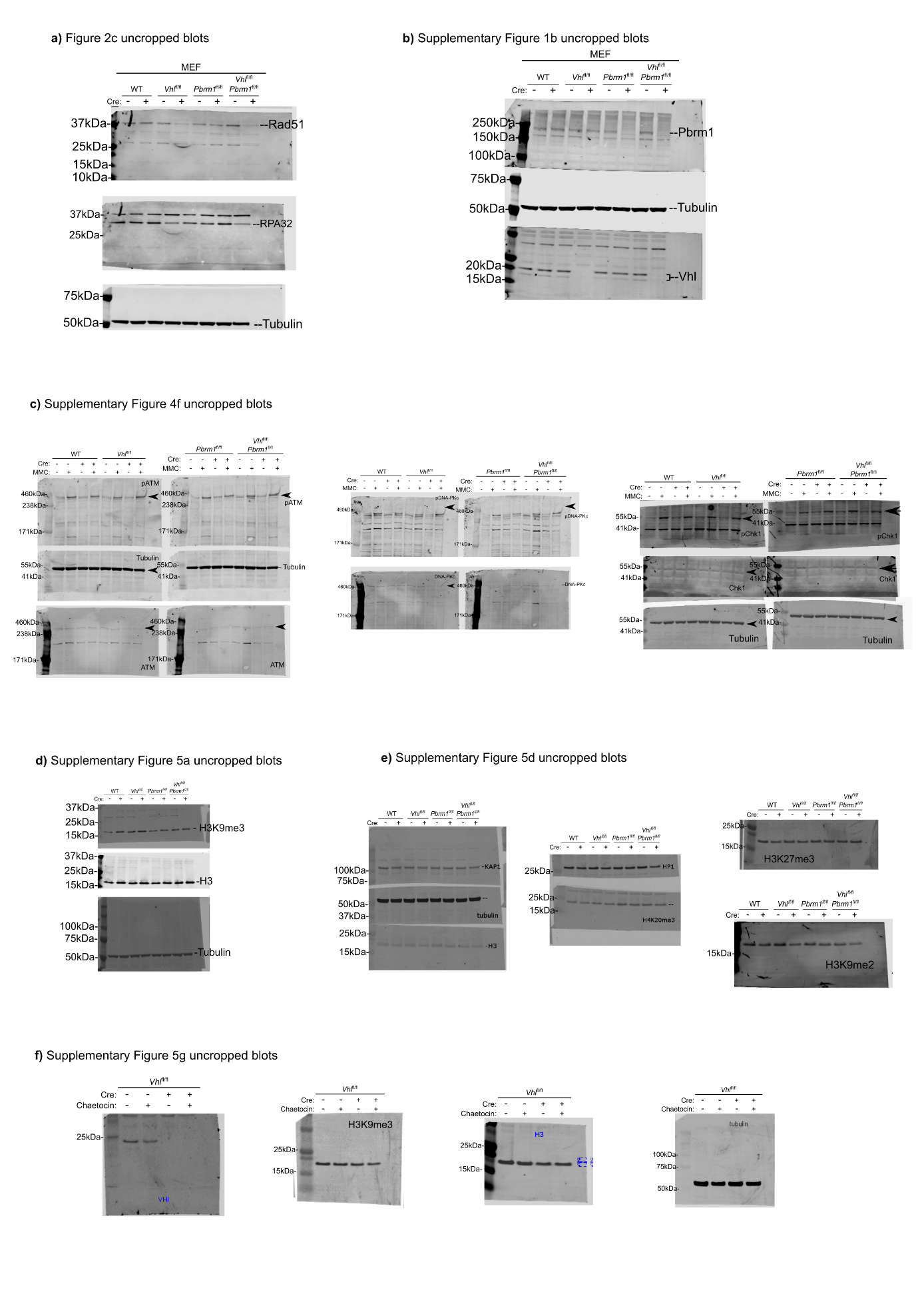


**Supplementary Figure 7. Uncropped blot images of western blots analyses presented in indicated figures.**
