# Supplementary material for: Loss of PBRM1 rescues VHL dependent replication stress to promote renal carcinogenesis
Source: Nat Commun. 2017 Dec 11;8:2026. doi: 10.1038/s41467-017-02245-1 (PMC5725450; doi:10.1038/s41467-017-02245-1)
Supplement: Supplementary file 1 — Peer Review File [file 41467_2017_2245_MOESM1_ESM.pdf]

**Editorial Note:** This manuscript has been previously reviewed at another journal that is not operating a transparent peer review scheme. This document only contains reviewer comments and rebuttal letters for versions considered at Nature Communications.
